# Short-term effects of occlusion therapy and optical correction on microvasculature in monocular amblyopia: a retrospective case–control study

**DOI:** 10.1038/s41598-023-38632-6

**Published:** 2023-07-27

**Authors:** Jae-Gon Kim, Se Youp Lee, Dong Cheol Lee

**Affiliations:** 1 Department of Ophthalmology, Keimyung University School of Medicine, Dongsan Medical Center, 1035 Dalgubeol-daero, Dalseo-gu, Daegu, 42601 Republic of Korea; 2 Graduate School of Medical Science and Engineering, Korea Advanced Institute of Science and Technology, Daejeon, 34141 Republic of Korea

**Keywords:** Anatomy, Medical research

## Abstract

This retrospective longitudinal case–control study investigated the short-term effects of patch occlusion treatment compared with optical correction on the microvasculature in monocular amblyopia. We included patients with monocular amblyopia treated for 2–12 months; they were classified into two groups according to the treatment regimen: patch occlusion or optical correction. Children aged < 12 years who presented to our clinic for examination without amblyopia diagnosis were enrolled as the control group. Changes in retinal and choroid microvasculature according to treatment were examined, and the correlation between changes in microvasculature and improvement in best-corrected visual acuity (BCVA) was evaluated. There were 57, 35, and 41 patients in the patch occlusion, optical correction, and control groups, respectively (mean age, 6.4 ± 2.0 years). Both amblyopic groups showed changes in the foveal and parafoveal deep capillary plexus vessel density (DCPD), choroidal thickness, and choroidal vascularity index (CVI) following short-term treatment (mean, 4.5 months). In the patch occlusion group, BCVA improved as the foveal DCPD increased (*P* = 0.013) and the CVI decreased (*P* = 0.037). In the optical correction group, BCVA improved as the foveal and parafoveal DCPD increased (*P* = 0.009). Increased foveal DCPD following amblyopia treatment and decreased CVI by patch occlusion were associated with improved BCVA.

## Introduction

Amblyopia is characterized by reduced best-corrected visual acuity (BCVA) in one or both eyes, compared to the normal BCVA in the eyes of the same age group, despite no structural abnormalities^1^.

The aim of occlusion treatment, as one of the treatment methods for amblyopia, is to stimulate the amblyopic eye and recover normal or close-to-normal visual function^1,2^. Change in BCVA is typically used to evaluate the effectiveness of occlusion treatment^2^; however, this is difficult in the case of young infants or children with cognitive impairments, such as intellectual disabilities or cerebral palsy.

Optical coherence tomography angiography (OCTA) can be used for patients who have difficulty in cooperating or concentrating, as it only requires the patient to stare at a single point of light for a short period of time to capture images. Until now, amblyopia has been known to interrupt the normal development of the cortical visual pathway^1^; however, structural abnormalities of the microvasculature in amblyopia have been reported recently after the development of OCTA^3–6^. The general consensus is that amblyopic eyes show reduced macular vessel density^3,6^ and increased choroidal thickness (CT^4,5^ compared with that noted in the fellow eye or in the control group. In addition, microvasculature of the retina and choroid is associated with low BCVA and changes in BCVA in patients with amblyopia^7–9^.

To date, it is presumed that patch occlusion restores vision via development of more extensive synaptic input to the visual cortex by stimulating the amblyopic eye^10,11^. Currently, there is limited knowledge about the structural changes that occur due to occlusion treatment. Recently, several studies have compared the microvasculatures of amblyopic and treated amblyopic eyes^8,12,13^. However, to the best of our knowledge, no study has compared pre- and post-patch occlusion treatment changes in the same eye and the patch occlusion-treated patients with optical correction-treated patients and normal controls. Additionally, previous studies have reported clinical improvement within 18 weeks after refractive error correction^2^ and within 10–17 weeks after initiating patch occlusion treatment^1^. However, little is known concerning the corresponding short-term changes in the retinal and choroidal microvasculature.

Several studies have established that visual stimulation can cause alterations in the microvasculature of the retina and choroid^14,15^. In addition, although the exact mechanism is not yet known, if the treatment of amblyopia affects the neural processing of visual information^10,11^, this could have downstream effects on the microvasculature of the retina and choroid. Changes in retinal function can ultimately affect the microvascular structure^16^. Therefore, we hypothesized that treatment-induced retinal or choroidal microvasculature changes could have upstream benefits on visual improvement.

This study aimed to determine the short-term changes in retinal and choroid microvasculature in amblyopic eyes following patch occlusion treatment by comparing them with changes in the eyes of patients who received only optical correction and of normal controls. Furthermore, it is necessary to confirm whether such changes correlate with the improvement of clinical symptoms.

## Methods

This retrospective longitudinal case–control study was approved on March 29, 2022, by the Keimyung University Dongsan Hospital Institutional Review Board (approval number: 2022-03-081). As this was a retrospective study, the Keimyung University Dongsan Hospital Institutional Review Board waived the requirement for informed consent. The study adhered to the tenets of the Declaration of Helsinki and followed all guidelines for experimental investigations in human participants.

This study was performed on patients aged < 12 years who were diagnosed with monocular amblyopia and underwent treatment for at least 2–12 months at most. Children with monocular amblyopia who visited Keimyung University Dongsan Hospital (Daegu, South Korea) from November 1, 2018, to September 31, 2021, were enrolled. Patients were divided into an optical correction group, in which only optical correction was performed, or a patch occlusion group, in which optical correction and patch occlusion were simultaneously performed. Those with binocular amblyopia were excluded from this study^1^. Patients who cooperated well with the ophthalmic examination and had good compliance with the treatment were included. Patients with deprivation amblyopia or ophthalmic diseases other than amblyopia or systemic diseases and those who underwent intraocular surgery were excluded from this study. Patients with spherical refractive errors > 5.00 diopter (D) and cylindrical refractive errors > 3.00 D were also excluded to avoid any effects on the OCTA measurements. Patients who presented to our clinic with BCVA that did not meet the diagnostic criteria for amblyopia and no ocular abnormality were enrolled in the control group. The amblyopic eyes of patients in the optical and patch occlusion groups were analyzed. In the control group, only the right eye was selected for analysis to remove the similarity of measures for the same person as a confounding factor.

### Diagnosis, treatment, and follow-up

Monocular amblyopia was diagnosed as a difference of ≥ 0.2 in BCVA by the logarithm of the minimum angle of resolution (logMAR) between the two eyes at baseline^1^. We assessed binocular alignment using the simultaneous prism cover/uncover test and examined ocular motility to confirm the presence of strabismus. The cycloplegic refraction was measured using 1% cyclopentolate hydrochloride eye drops and retinoscopy^2^. If anisohyperopia ≥ 1.5 D or anisomyopia ≥ 2.5 D was detected through cycloplegic refraction, we diagnosed the patient with anisometropic amblyopia^17^. Patients with anisometropic amblyopia and ocular deviation on the simultaneous prism cover/uncover test were diagnosed with mixed amblyopia; thus, only those with anisometropic and mixed (strabismic and anisometropic) amblyopia were included^2^.

Optical correction was prescribed for all patients based on the results of cycloplegic refraction at baseline, which followed the Preferred Practice Pattern guidelines published by the American Academy of Ophthalmology in 2018^17^. At our hospital, some parents wanted to proceed with refractive correction and patch occlusion simultaneously in anticipation of faster improvement. Patch occlusion treatment was started at 2 h/day for mild to moderate amblyopia (> 20/80) and 6 h/day for severe amblyopia (< 20/80).

Follow-up was scheduled 2 months after starting treatment in all patients. However, if a patient missed their scheduled visit or if OCTA images were taken on time but were of inadequate quality to measure all parameters, we used the date of the subsequent clear image as the “Follow-up” date. The patch occlusion time was adjusted based on the patient’s response, with an increase to 4–6 h/day if there was no improvement in BCVA at the next visit and a decrease to 2–4 h/day if there was an improvement.

### Clinical data collection

Cycloplegic refraction was recorded as spherical equivalent at baseline and the first follow-up. Stereo Fly stereoscopic tests were conducted to measure stereoscopic acuity. The average patching time from baseline to follow-up was recorded for each patient. Owing to the retrospective design of this study, the axial lengths of the patients were not recorded. However, considering previous studies showing that axial length affected the macular microvasculature and CT^18,19^, we calculated an approximation of the axial length using age, sex, and spherical equivalent to adjust for statistical analysis^20–24^.

### OCTA data collection

**Figure 1:**
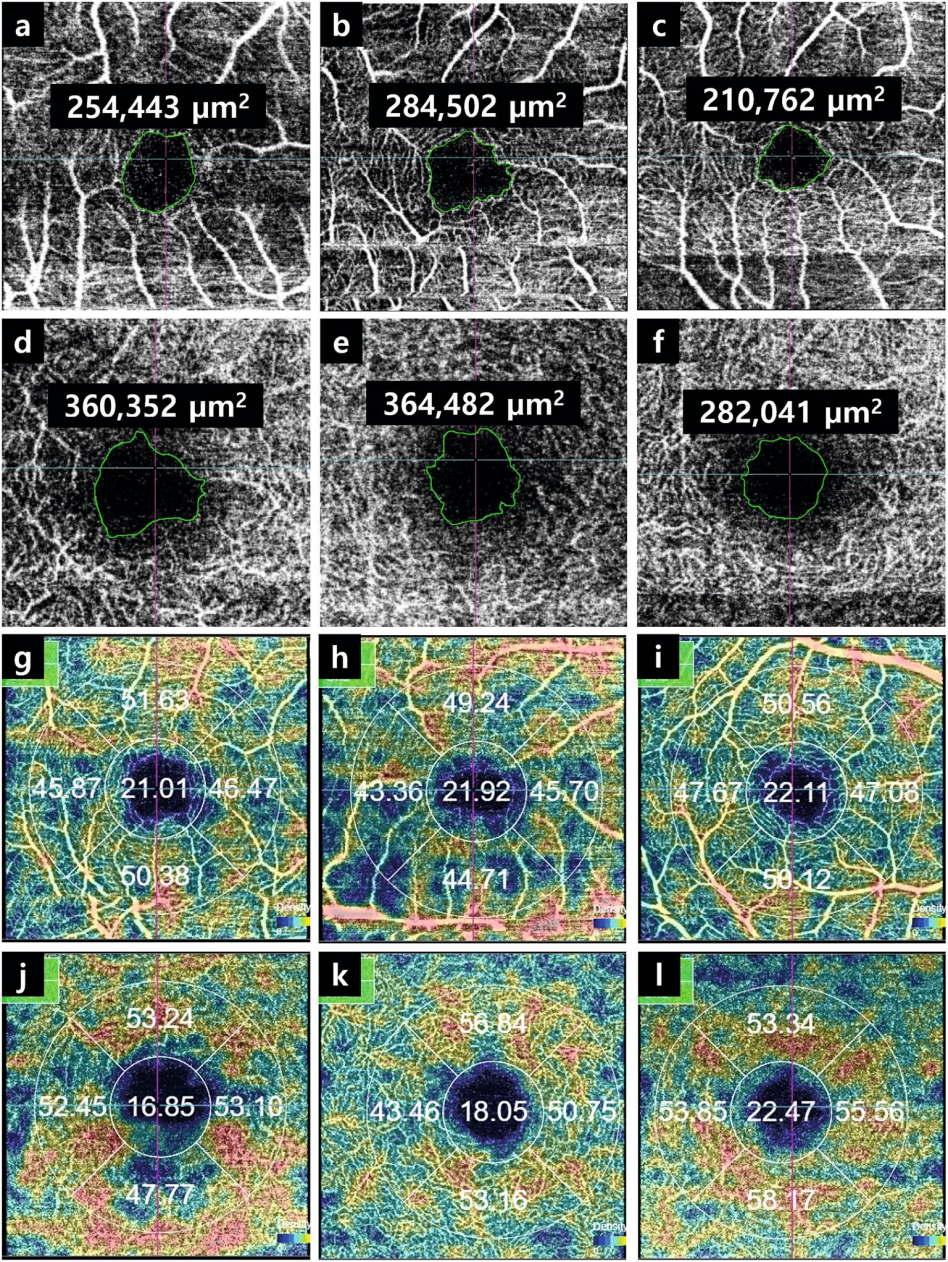
Representative images of retinal microvasculature measured using OCTA. (**a**–**c**) SFAZ area and (**d**–**f**) DFAZ area; the dark avascular area in both the SCP and DCP was manually measured using the IMAGEnet program by outlining its boundary. (**g**–**i**) SCPD and (**j**–**l**) DCPD; vessel density in the SCP and DCP layers were automatically calculated using IMAGEnet. The central number was recorded as either foveal SCPD or DCPD. The parafovea was segmented into four regions, namely top, bottom, temporal, and nasal, and the mean vessel density of each region was computed and noted as parafoveal SCPD and DCPD (**a**, **d**, **g**, **j**: patch occlusion group; **b**, **e**, **h**, **k**: optical correction group; **c**, **f**, **i**, **l**: control group). The images were created using the built-in software program IMAGEnet6 (version 1.25.16650; Topcon, URL: https://topconhealthcare.jp/products/imagenet-6/) in the DRI OCT Triton Plus instrument (Topcon Co., Tokyo, Japan). OCTA, optical coherence tomography angiography; SFAZ, superficial retinal capillary plexus foveal avascular zone; DFAZ, deep retinal capillary plexus foveal avascular zone; SCPD, vessel density of the superficial retinal capillary plexus; DCPD, vessel density of the deep retinal capillary plexus.

**Figure 2:**
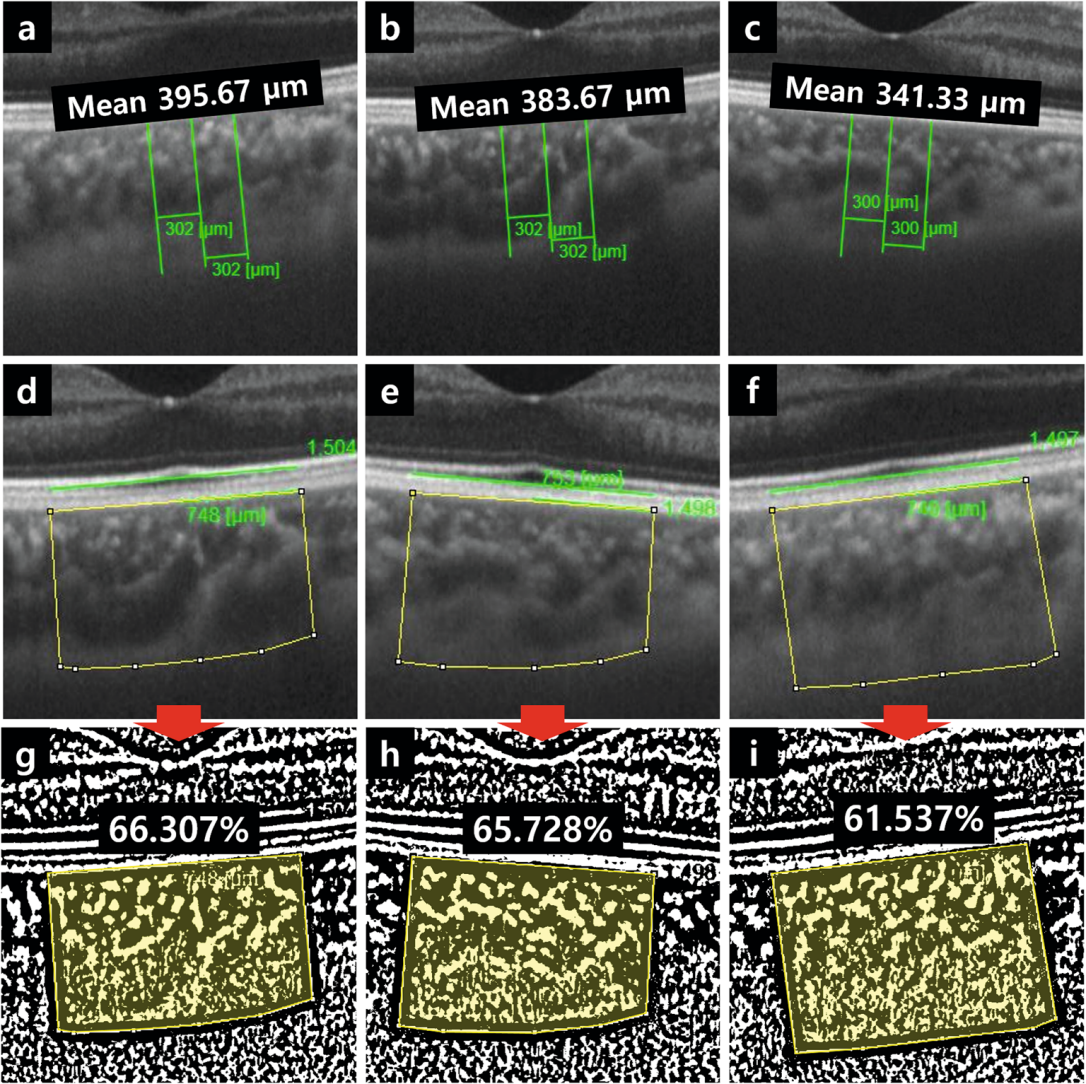
Representative images of choroidal microvasculature measured using OCTA. (**a**–**c**) Using the IMAGEnet program, CT was manually measured from the start of the choriocapillary layer just beneath the retinal pigment epithelial-Bruch's membrane complex to the choroid-sclera boundary, relative to the macular surface. The measurement was repeated at a location 300 µm to the nasal and temporal side of the macula, and the average of all three measurements was used. (**d**–**i**) CVI was manually measured using ImageJ. A 1500 µm horizontal length of the choroid was used as the reference, covering 750 µm on both the nasal and temporal sides of the macula (which was demarcated by a yellow border). The image was converted to a binary format using Niblack's method in ImageJ (**g**–**i**). The ratio of black pixels (vascular area) to the sum of black and white pixels (total area) inside the yellow area was calculated (**a**, **d**, **g**: patch occlusion group; **b**, **e**, **h**: optical correction group; **c**, **f**, **i**: control group). The images were created using the built-in software program IMAGEnet6 (**a**–**f**) (version 1.25.16650; Topcon, URL: https://topconhealthcare.jp/products/imagenet-6/) in the DRI OCT Triton Plus instrument (Topcon Co., Tokyo, Japan), and processed using the ImageJ program (**g**–**i**) (version 1.52a; National Institutes of Health, Bethesda, MD, URL: https://imagej.nih.gov/ij/download.html). OCTA, optical coherence tomography angiography; CT, choroidal thickness; CVI, choroidal vascularity index.

OCTA was performed for all included patients using a spectral-domain device (DRI OCT Triton Plus; Topcon, Tokyo, Japan) at baseline and follow-up. The parameters of the flow areas in the fovea-centered 6 × 6 mm scan size were measured using built-in optical software (IMAGEnet 6, version 1.25.16650; Topcon). The collected data included the area of the superficial retinal capillary plexus foveal avascular zone (SFAZ) and deep retinal capillary plexus foveal avascular zone (DFAZ); vessel density of the superficial retinal capillary plexus (SCPD), vessel density of the deep retinal capillary plexus (DCPD), CT, and choroidal vascularity index (CVI) at baseline and follow-up. The SFAZ area, DFAZ area, and CT were measured manually by the investigator using IMAGEnet, and the CVI, which is the percentage of luminal area occupied by blood vessels within a certain choroidal area, was measured manually using ImageJ (version 1.52a; National Institutes of Health, Bethesda, MD) **(**Figs. 1a–f and 2). Vessel density, the percentage of area occupied by blood vessels within a certain macular area, was calculated automatically using IMAGEnet. Vessel density was divided into SCPD and DCPD and also into “foveal vessel density,” measured inside a circle with a diameter of 1 mm centered at the fovea, and “parafoveal vessel density,” which is an average density within an outer circle ranging from 1 to 3 mm in diameter (Fig. 1g–l). The calculated axial length and follow-up period were considered potential confounding factors.

### Statistical analyses

The total sample size was calculated using G*Power 3.1.9.2. (University of Dusseldorf, Dusseldorf, Germany), and 108 participants were needed to account for 95% power, a statistical level of significance of 0.05, and an effect size of 0.25. The baseline characteristics of each group were analyzed using traditional descriptive methods, one-way analysis of variance (ANOVA), and Pearson’s chi-squared test. Functional parameters were compared between the groups using one-way ANOVA, and post-hoc testing was performed for variables showing significant differences using the Games–Howell test. OCTA parameters were compared between the groups using a one-way analysis of covariance (ANCOVA), which was adjusted for axial length at each time point. To test whether the statistical significance was maintained after adjusting for the effects of axial length and follow-up duration, we conducted statistical analysis by inserting both variables as covariates^25^. A post-hoc test was performed using Bonferroni correction. Changes in functional parameters were compared using a paired t-test. Changes in OCTA parameters were analyzed by repeated-measures ANCOVA, adjusted for the baseline axial length and the follow-up period. Multivariate linear regression analysis with baseline axial length adjustment was performed on the OCTA parameters that significantly changed in the patch occlusion and optical correction groups to analyze whether the amount of change in each parameter was related to the change in BCVA and stereopsis. Since the number of people in each group exceeded 30, normality was considered satisfied. Analyses were performed using SPSS Statistics 25.0.0 (IBM, Armonk, NY). A two-tailed *P*-value ≤ 0.05 was considered statistically significant.

## Results

Overall, 133 participants were included in this study. Among them, 57 patients were in the patch occlusion group, 35 were in the optical correction group, and 41 children were in the control group. The mean age and follow-up period were not significantly different between the three groups (*P* = 0.126 and 0.611). The mean daily patch occlusion treatment time was 2.8 ± 1.0 (range 2–6) h/day. Patch occlusion treatment was administered for an average of 2 h/day in 30 patients, ranging from 2 to 6 h/day in 26 patients, with one patient receiving 6 h/day of treatment. At baseline, 77 patients were diagnosed with anisometropic amblyopia, and 15 were diagnosed with mixed amblyopia of the total patients. There were no significant differences in amblyopia composition between groups (*P* = 0.681). The baseline characteristics of the patients are shown in Table 1.

**Table 1:** Demographics and clinical characteristics.

Characteristics of patients		Patch occlusion	Optical correction	Control (right eye)	*P*-value
Total number of eyes enrolled (patients)		57	35	41	–
Age (years)		6.5 ± 1.9	5.8 ± 1.7	6.7 ± 2.3	0.126
Sex (male : female)		34 : 23	17 : 18	20 : 21	0.455
Mean follow-up period (months)		4.2 ± 2.9	4.8 ± 2.7	4.5 ± 2.3	0.611
Mean daily patching time (h/day)		2.8 ± 1.0	–	–	–
Diagnosis	Anisometropic	47	30	–	0.681
	Mixed	10	5	–	

Data are presented either as the mean ± standard deviation or as numerical values. *P*-values compared between the three groups were calculated by analysis of variance, and categorical variables (sex, diagnosis) were assessed using Pearson’s chi-squared test. *P < 0.05.

### Comparison at baseline

No significant differences were noted in functional parameters between the patch occlusion and optical correction groups. Similarly, no significant difference was noted in baseline BCVA between the patch occlusion and the optical correction groups (*P* = 0.529). The DFAZ area was wider in the optical correction group than in the control group (*P* = 0.011). Similarly, parafoveal DCPD was also lower in only the optical correction group (*P* = 0.036) than in the control group. Foveal DCPD was lower in both the amblyopic groups than in controls (*P* = 0.033 and 0.042), CT was increased (*P* = 0.010 and 0.018), and CVI was higher (*P* = 0.014 and 0.012). No significant differences were noted in OCTA parameters between the patch occlusion and optical correction groups. Comparisons of functional and OCTA parameters at baseline are summarized in Supplementary Table S1.

### Effects of treatment

After treatment, foveal DCPD increased (*P* = 0.017), parafoveal DCPD increased (*P* = 0.036), CT decreased (*P* = 0.019), and CVI decreased (*P* = 0.018) in the patch occlusion group. In the optical correction group, foveal DCPD increased (*P* = 0.032), parafoveal DCPD increased (*P* = 0.019), CT decreased (*P* = 0.024), and CVI decreased (*P* = 0.026). In the control group, no significant microvasculature changes were noted. Changes in the functional and OCTA parameters from baseline to follow-up in each group are summarized in Supplementary Table S2.

### Comparison at follow-up

When the axial length at follow-up was adjusted, the DFAZ area, foveal DCPD, parafoveal DCPD, CT, and CVI, which showed differences between the groups at baseline, did not show a significant difference. A comparison of the functional and OCTA parameters at follow-up is summarized in Supplementary Table S1.

### Multivariate analysis

In the patch occlusion group, when foveal DCPD was increased by 1%, BCVA decreased by 0.320 logMAR (*P* = 0.013) and when CVI was decreased by 1%, BCVA decreased by 0.273 logMAR (*P* = 0.037). In the optical correction group, when foveal DCPD was increased by 1%, BCVA decreased by 0.442 logMAR (*P* = 0.009), showing the same trend as that in the patch occlusion group. None of the variables showed a significant correlation with the change in stereopsis in each group. The results of the multivariate linear regression analysis are shown in Table 2.

**Table 2:** Multivariate linear regression analysis of the association between changes in BCVA and stereopsis and changes in other variables.

	Patch occlusion				Optical correction			
	BCVA		Stereopsis		BCVA		Stereopsis	
	Standardized ß coefficient	*P*-value	Standardized ß coefficient	*P*-value	Standardized ß coefficient	*P*-value	Standardized ß coefficient	*P*-value
Foveal DCPD (%)	− 0.320	0.013*	− 0.170	0.210	− 0.442	0.009*	0.162	0.340
Parafoveal DCPD (%)	0.015	0.908	0.084	0.542	0.268	0.126	− 0.150	0.374
CT (μm)	− 0.140	0.289	− 0.246	0.068	− 0.226	0.220	0.103	0.564
CVI (%)	0.273	0.037*	− 0.153	0.265	− 0.048	0.785	0.144	0.393

Changes in variables were calculated as follow-up minus baseline.* P*-values were calculated using multivariate linear regression analysis adjusted for baseline axial length. *P < 0.05.

BCVA, best-corrected visual acuity; DCPD, deep capillary plexus density; CT, choroidal thickness; CVI, choroidal vascularity index.

## Discussion

Our study illustrated changes in microvasculature according to the treatment of amblyopia. In particular, increased foveal DCPD was associated with improvement in BCVA in patients who underwent optical correction, and a decrease in CVI was also associated with improvement in BCVA when the patch occlusion was used together.

Various previous studies have reported differences between the retinal and choroidal microvasculature in amblyopic and normal eyes^5,6,26–29^. In this study, the DFAZ area of the amblyopic eye was wider than that of the normal control eye. In fact, the results of studies related to FAZ in amblyopic eyes are controversial^6^. Among them, in the study by Sobral et al*.*^29^, the DFAZ area was wider in amblyopic eyes than in the control eyes, similar to the results of the present study. In contrast, both SCPD and DCPD were generally lower in amblyopic eyes than in the control eyes^6^. However, no significant difference was noted in SCPD in this study, and only DCPD was lower in the parafovea in amblyopic eyes than in control eyes. The study by Demirayak et al*.*^28^ on adult patients with amblyopia showed similar results to those of this study. CT has been reported to be higher in the amblyopic eye than in the normal control eye^5^, and the CVI was higher in the amblyopic eye than in the normal control eye^26,27^, which is consistent with the results of this study.

Several studies have also investigated changes in the microvasculature of the retina and choroid associated with the treatment of amblyopia^8,9,12,30^. Among them, a significant decrease in CT after amblyopia treatment was reported by Aslan Bayhan and Bayhan^12^. Furthermore, Nishi et al*.*^9^ found that the CVI of the amblyopic eye was decreased with treatment, approaching that of the normal control eye. The results of these studies are in line with our findings.

In this study, foveal and parafoveal DCPD were increased, and CT and CVI were decreased in amblyopic eyes according to treatment in both groups. Amblyopic eyes showed changes in DCPD and choroid in the outer retina rather than the inner retina. Based on these results, we hypothesized that the occurrence and recovery of amblyopia are related to the outer retina, where the DCP is located, and the choroid rather than the inner retina. Synapses between photoreceptor terminals and bipolar cells occur in areas where the DCP is located^31^. Lower DCPD may be related to decreased synaptic interactions^28^. As the treatment progresses, synapses may form, and DCPD may increase in amblyopic eyes, and accordingly, CT and CVI, which increase in compensation^5,27^, are also considered to be normalized.

Through multivariate analysis, we found that changes in foveal DCPD were positively associated with improvement in BCVA in both groups. Several studies have found that improvement in or worsening of DCPD is associated with better or worse visual acuity, respectively^32,33^. These studies have explained that this association is attributed to the change in the number of synapses in the outer plexiform layer. That is, the number of synapses between photoreceptors with horizontal and bipolar cells changes, these synapses receive blood supply from the DCP, and a series of processes are related to vision^29,33^. Another explanation is that because the DCP provides photoreceptors with approximately 15% of their oxygen supply, its changes can affect the function of photoreceptors^32,33^. Whether the photoreceptors are involved in amblyopic development remains controversial. Al-Haddad et al*.*^30^ suggested several signs of foveal immaturity in the amblyopic eye, implying that photoreceptors may be abnormal due to amblyopic progression. Therefore, we presume that as amblyopia treatment progresses, the synaptic connections in the outer plexiform layer increase, and the photoreceptor function improves, improving BCVA. However, unlike foveal DCPD, parafoveal DCPD was not associated with improvement in BCVA, although there was a significant change compared to the baseline. It can be assumed that parafoveal DCPD did not change enough to affect visual acuity after a short-term treatment of 4.5 months or that amblyopia recovery was more related to cone cells rather than rod cells. Indeed, when the average changes in foveal and parafoveal DCPD in the two amblyopic groups were compared, foveal DCPD increased by 1.03% more than did parafoveal DCPD.

Unlike in the optical correction group, the decrease in CVI was correlated with improvement in BCVA only in the patch occlusion group. According to previous studies^26,27^, CVI is thought to compensate for the increased blood supply to the outer retina in amblyopic eyes and decreases with treatment^9^. Under dark conditions, choroidal blood flow decreases, and unilateral light blocking affects choroidal blood flow in both eyes^34,35^. During patch occlusion treatment, the light in the fellow eye is blocked; thus, the choroid blood flow in the amblyopic eye might decrease. As this is repeated, the extended choroidal vessel in the amblyopic eye may contract, and the CVI decreases. The amblyopic eye may also be sensitive to visual stimulation, and the retinal vessels and visual cortex may develop better, improving visual acuity. Second, the stromal area increases when the luminal area decreases during patch occlusion treatment. Furthermore, there are non-vascular smooth muscle cells in the stromal area^36^ that have been reported to be related to accommodation^37^, and accommodation is reduced in amblyopic eyes compared to fellow eyes^38^. During patch occlusion treatment, the number of non-vascular smooth cells in the stromal area may increase, and accommodation may improve in the amblyopic eye. Improvements in accommodation could induce the development of visual acuity^38^.

The strength of this study lies in its investigation of the longitudinal impact of amblyopia treatment on both retinal and choroidal microvasculature, unlike previous studies that have focused solely on the effects of amblyopia treatment on retinal microvasculature^39,40^. Moreover, instead of simply comparing the amblyopia and control groups, this study compared patients undergoing optical correction with those undergoing patch occlusion treatment, thus examining the exclusive effects of patch occlusion treatment. In addition, this study not only assessed the changes in microvasculature before and after treatment but also explored the correlations between these changes and improvements in BCVA. Our findings substantiate that reduction in CVI through patch occlusion treatment was associated with enhancements in BCVA.

Nevertheless, further prospective studies with larger sample sizes are required to validate our findings. Our retrospective design may have caused selective bias. As this study relied on medical records, the patients' follow-up periods differed. Although it was adjusted in consideration of the effect of the change in OCTA parameters, the potential impact of changes in treatment compliance throughout follow-up was not considered in this study. Thus, it is necessary to control the follow-up period in further studies. Moreover, we were unable to perform axial length measurements. Although all comparisons were made with calculated axial length using spherical equivalent, age, and sex as covariates, it is necessary to examine the actual axial length in a later study.

In conclusion, in this short-term retrospective longitudinal study, we found that changes in foveal DCPD following treatment were linearly correlated with improvement in BCVA. Particularly, reduction in CVI following patch occlusion treatment was linearly correlated with improvement in BCVA. We hypothesized that this might be related to changes in choroidal microvasculature in the amblyopic eye due to light blocking in the fellow eye during patch occlusion treatment. These characteristics that appear only in patch occlusion treatment are expected to be helpful in indirectly determining the improvement in visual acuity following this treatment.

## Supplementary Information

Supplementary Tables.

## Data Availability

The datasets used and/or analyzed during the current study are available from the corresponding author upon reasonable request.
