# Supplementary material for: Short-term effects of occlusion therapy and optical correction on microvasculature in monocular amblyopia: a retrospective case–control study
Source: Sci Rep. 2023 Jul 27;13:12191. doi: 10.1038/s41598-023-38632-6 (PMC10374566; doi:10.1038/s41598-023-38632-6)
Supplement: Supplementary file 1 — Supplementary Tables. [file 41598_2023_38632_MOESM1_ESM.pdf]

Short-term effects of occlusion therapy and optical correction on microvasculature in monocular amblyopia: a retrospective case-control study

Jae-Gon Kim, Se Youp Lee, Dong Cheol Lee*

Supplementary Information

Supplementary Table S1. Comparison of functional and OCTA parameters between groups at baseline and follow-up

Baseline	Patch occlusion	Optical correction	Control (right eye)	*P*-value	Post hoc (*P*-value)
BCVA (logMAR)	0.414±0.196	0.371±0.176	0.099±0.082	<0.001[a]*	P(<.001[c]*)O(<.001[c]*)>C
Stereopsis (seconds of arc)	758.8±1240.6	620.7±1090.3	52.7±38.9	0.002[a]*	P(<.001[c]*)O(.011[c]*)>C
Spherical equivalent (D)	2.428±3.533	1.893±3.157	0.034±1.012	<0.001[a]*	P(<.001[c]*)O(.005[c]*)>C
Axial length (mm)	22.058±1.716	22.153±1.709	23.177±0.773	0.001[a]*	P(<.001[c]*)O(.006[c]*)>C
SFAZ area (μm²)	254 256.8±111 033.0	257 893.1±91 495.1	225 493.1±116 574.3	0.603[b]	
DFAZ area (μm²)	320 142.4±115 654.3	375 923.2±164 628.7	281 671.0±124 145.0	0.013[b]*	P(.557[d])O(.011[d]*)>C
Foveal SCPD (%)	23.958±5.503	23.744±5.615	24.664±4.901	0.716[b]	
Parafoveal SCPD (%)	47.299±2.590	47.888±2.750	48.616±2.398	0.162[b]	
Foveal DCPD (%)	18.018±4.654	17.816±4.358	21.595±8.227	0.018[b]*	P(.033[d]*)O(.042[d]*)<C
Parafoveal DCPD (%)	51.228±3.150	50.669±3.658	52.916±3.077	0.033[b]*	P(.147[d])O(.036[d]*)<C
CT (μm)	384.0±60.0	385.2±59.6	349.9±49.3	0.006[b]*	P(.010[d]*)O(.018[d]*)>C
CVI (%)	65.090±2.925	65.314±2.641	63.446±2.875	0.005[b]*	P(.014[d]*)O(.012[d]*)>C
Follow-up					
BCVA (logMAR)	0.248±0.211	0.236±0.227	0.076±0.061	<0.001[a]*	P(<0.001[c]*)O(0.001[c]*)>C
Stereopsis (seconds of arc)	387.9±747.6	312.9±697.0	53.2±51.8	0.026[a]*	P(0.004[c]*)O(0.086[c])>C
Spherical equivalent (diopter)	2.252±3.635	1.657±3.112	-0.122±1.175	0.001[a]*	P(<0.001[c]*)O(0.007[c]*)>C
Axial length (mm)	22.258±1.750	22.352±1.685	23.328±0.773	0.002[a]*	P(<0.001[c]*)O(0.008[c]*)<C
SFAZ area (μm²)	239 566.2±118 822.7	255 021.4±88 641.4	216 007.3±123 017.1	0.436[b]	
DFAZ area (μm²)	312 295.4±119 731.8	353 822.5±120 936.2	277 775.1±142 746.3	0.075[b]	

Foveal SCPD (%)	24.643±5.326	23.221±5.399	24.177±4.016	0.410[b]	
Parafoveal SCPD (%)	47.446±2.593	48.042±2.645	48.810±2.829	0.062[b]	
Foveal DCPD (%)	19.283±5.395	18.968±5.171	22.873±10.774	0.110[b]	
Parafoveal DCPD (%)	52.254±3.001	51.708±4.097	52.523±4.258	0.661[b]	
CT (μm)	368.5±64.2	369.3±60.8	358.9±61.9	0.458[b]	
CVI (%)	63.794±3.062	64.030±3.112	62.600±2.326	0.109[b]	

Values are presented as means±standard deviations or number. *P*-values were calculated using [a] analysis of variance or [b] analysis of covariance adjusted for axial length measured at each time point. *P*-values of the post-hoc test were calculated using [c] Games–Howell test or [d] Bonferroni correction.[*] $P<0.05$

OCTA, optical coherence tomography angiography; BCVA, best-corrected visual acuity; LogMAR, logarithm of the minimum angle of resolution; P, patch occlusion group; O, optical correction group; C, control group; SFAZ, superficial foveal avascular zone; DFAZ, deep foveal avascular zone; SCPD, superficial capillary plexus density; DCPD, deep capillary plexus density; CT, choroidal thickness; CVI, choroidal vascularity index.

Supplementary Table S2. Changes in functional and OCTA parameters from baseline to follow-up

	Patch occlusion			Optical correction			Control (right eye)		
	Baseline	Follow-up	*P*-value	Baseline	Follow-up	*P*-value	Baseline	Follow-up	*P*-value
BCVA (logMAR)	0.414±0.196	0.248±0.211	<0.001[a]*	0.371±0.176	0.236±0.227	<0.001[a]*	0.099±0.082	0.076±0.061	0.031[a]*
Stereopsis (seconds of arc)	758.8±1240.6	387.9±747.6	0.002[a]*	620.7±1090.3	312.9±697.0	0.008[a]*	52.7±38.9	53.2±51.8	0.938[a]
Spherical equivalent (D)	2.428±3.533	2.252±3.635	0.051[a]	1.893±3.157	1.657±3.112	0.062[a]	0.034±1.012	-0.122±1.175	0.214[a]
Axial length (mm)	22.058±1.716	22.258±1.750	<0.001[a]*	22.153±1.709	22.352±1.685	0.002[a]*	23.177±0.773	23.328±0.773	0.026[a]*
SFAZ area (μm^2)	254,256.8±111,033.0	239,566.2±118,822.7	0.302[b]	257,893.1±91,495.1	255,021.4±88,641.4	0.838[b]	225,493.1±116,574.3	216,007.3±123,017.1	0.237[b]
DFAZ area (μm^2)	320,142.4±115,654.3	312,295.4±119,731.8	0.594[b]	375,923.2±164,628.7	353,822.5±120,936.2	0.394[b]	281,671.0±124,145.0	277,775.1±142,746.3	0.779[b]
Foveal SCPD (%)	23.958±5.503	24.643±5.326	0.396[b]	23.744±5.615	23.221±5.399	0.643[b]	24.664±4.901	24.177±4.016	0.581[b]
Parafoveal SCPD (%)	47.299±2.590	47.446±2.593	0.605[b]	47.888±2.750	48.042±2.645	0.746[b]	48.616±2.398	48.810±2.829	0.742[b]
Foveal DCPD (%)	18.018±4.654	19.283±5.395	0.017[b]*	17.816±4.358	18.968±5.171	0.032[b]*	21.595±8.227	22.873±10.774	0.509[b]
Parafoveal DCPD (%)	51.228±3.150	52.254±3.001	0.036[b]*	50.669±3.658	51.708±4.097	0.019[b]*	52.916±3.077	52.523±4.258	0.608[b]
CT (μm)	384.0±60.0	368.5±64.2	0.019[b]*	385.2±59.6	369.3±60.8	0.024[b]*	349.9±49.3	358.9±61.9	0.182[b]
CVI (%)	65.090±2.925	63.794±3.062	0.018[b]*	65.314±2.641	64.030±3.112	0.026[b]*	63.446±2.875	62.600±2.326	0.095[b]

Values are presented as means±standard deviations or numbers. [a] paired t-test or [b] repeated-measures analysis of covariance adjusted for baseline axial length and follow-up period. [*] *P*<0.05

OCTA, optical coherence tomography angiography; BCVA, best-corrected visual acuity; LogMAR, logarithm of the minimum angle of resolution; SFAZ, superficial foveal avascular zone; DFAZ, deep foveal avascular zone; SCPD, superficial capillary plexus density; DCPD, deep capillary plexus density; CT, choroidal thickness; CVI, choroidal vascularity index.
